# Increased gene dosage for β- and κ-casein in transgenic cattle improves milk composition through complex effects

**DOI:** 10.1038/srep37607

**Published:** 2016-11-23

**Authors:** Götz Laible, Grant Smolenski, Thomas Wheeler, Brigid Brophy

**Affiliations:** 1 AgResearch, Ruakura Research Centre, Hamilton, New Zealand

## Abstract

We have previously generated transgenic cattle with additional copies of bovine *β-* and *κ casein* genes. An initial characterisation of milk produced with a hormonally induced lactation from these transgenic cows showed an altered milk composition with elevated β-casein levels and twofold increased κ-casein content. Here we report the first in-depth characterisation of the composition of the enriched casein milk that was produced through a natural lactation. We have analyzed milk from the high expressing transgenic line TG3 for milk composition at early, peak, mid and late lactation. The introduction of additional *β-* and *κ-*casein genes resulted in the expected expression of the transgene derived proteins and an associated reduction in the size of the casein micelles. Expression of the transgenes was associated with complex changes in the expression levels of other milk proteins. Two other major milk components were affected, namely fat and micronutrients. In addition, the sialic acid content of the milk was increased. In contrast, the level of lactose remained unchanged. This novel milk with its substantially altered composition will provide insights into the regulatory processes synchronizing the synthesis and assembly of milk components, as well as production of potentially healthier milk with improved dairy processing characteristics.

The composition of milk has been optimized through the evolution of mammals to provide an optimal balance of protein, lipids and carbohydrate to support the growth and wellbeing of the newborn offspring^1^. The most abundant proteins in milk, the caseins, are packaged into an ordered micelle, which facilitates an extremely high protein concentration, 30 mg/ml for cows’ milk, while maintaining low viscosity and high solubility. Similarly, the triacylglycerides in milk are packaged into a phospholipid membrane-bound globule^2^, and many of the minerals and other micronutrients in milk are complexed to carrier proteins^3^. The microstructures and complexes within milk are important for its dairy processing characteristics^4^,^5^ and presumably also its biological functions.

Uniquely among mammals, humans consume milk from other species, as a major food source. However, cows’ milk has a distinct protein repertoire compared with human milk^6^, and the protein content of human milk is lower and the lactose content is higher compared with cows’ milk^7^. Differences in viscosity and micelle characteristics also exist^8^,^9^ and micelle size has been shown to affect the processing properties of milk^10^. Thus there is the potential to alter the composition of cows’ milk so as to better optimize its biological functionality as well as its processing characteristics as a food for the human population. The potential of a transgenic approach for improving the biological function of milk for human nutrition has so far been mainly investigated through boosting the antimicrobial characteristics of milk by expressing human lactoferrin and lysozyme in the milk of dairy animals^11^. Other attempts have been targeted at milkfat in order to reduce the saturated fatty acid content of milk. A diet high in saturated fats has been linked with an increased risk of cardiovascular and coronary heart disease in humans and expression of desaturases were used as strategy to decrease the ‘unhealthy’ milkfats in favour of healthier unsaturated fats^12^,^13^,^14^,^15^.

We have previously reported the generation of dairy cows expressing additional β- and κ-casein genes in their mammary glands and demonstrated secretion of the transgene-derived caseins into milk upon artificial hormonal stimulation of the mammary gland^16^. The mammary secretions from the control and transgenic lines appeared to have distinct characteristics. However, the small quantities of secretion obtainable by inducing milk secretion artificially before puberty through the application of a hormonal treatment regime, precluded an in-depth analysis of its composition. In a previous study, we have reported on the processing of the milk from these transgenic cows into cheese^17^. Here we report the first analysis of the effects of the expression of the additional milk protein genes on the abundance and degree of post-translational modification of individual milk proteins, micronutrient composition, and micelle characteristics in milk from a naturally induced lactation following calving. The results show that expression of the additional casein genes results in several changes, some of which have potential relevance for human health or dairy processing.

## Results

### Transgene-derived caseins are expressed in natural lactation milk

**Figure 1:**
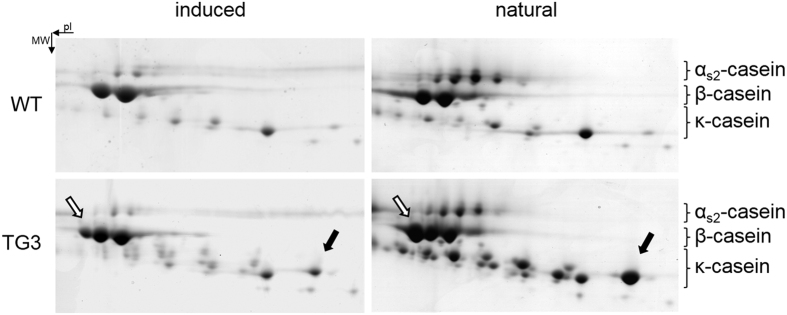
Two-dimensional electrophoresis of mammary secretion from a transgenic (TG3) and a control (WT) cow. Mammary secretion was collected from a TG3 and a wild type cow, firstly as one-year old heifers after hormonal induction of lactation (induced), and then from the same animals at peak lactation after a natural calving (natural). Milk proteins were separated by isoelectric point (pI) in the first dimension and molecular weight (MW) in the second dimension. The gels were stained for protein with Coomassie blue and sections of each gel are shown in which the major caseins (as marked) are resolved. The white centred arrow indicates the exogenously expressed A3 isoform of β-casein. The black centred arrow indicates the exogenously expressed B isoform of κ-casein. Shown are representative gels which have been cropped for improved clarity. The full size gels are presented in Supplementary Fig. S2.

We have previously reported the generation of the transgenic cattle line TG3, harbouring additional genes for bovine β- and κ-casein and described their expression potential deduced from a hormonally induced lactation^16^. To confirm that the transgene derived milk proteins β-casein A3 and κ-casein B are expressed in natural lactation milk at similar levels to what we have detected in hormonally induced milk we initially analyzed early lactation milk samples from the first natural lactation of seven TG3 founder cows and three wild type control cows with the same genetic background. Two-dimensional (2D) analysis of milk proteins revealed a similar pattern of relative abundance for induced and natural milk (Fig. 1). The transgene derived β-casein A3 and κ-casein B variants were enhanced in abundance in natural transgenic milk compared to induced transgenic milk. In contrast to induced TG3 milk, in natural TG3 milk the transgene-derived caseins were the most highly expressed variants of β-casein and κ-casein, well above the expression levels of the endogenous variants β-casein A1 and A2 and κ-casein A. In addition, the relatively high abundance of κ-casein B resulted in a more pronounced representation of the glycosylated κ-casein isoforms in the natural milk sample from the TG3 cows compared to the induced milk.

### Effect of the increased gene dosage for β- and κ- casein on their expression levels in milk

**Figure 2:**
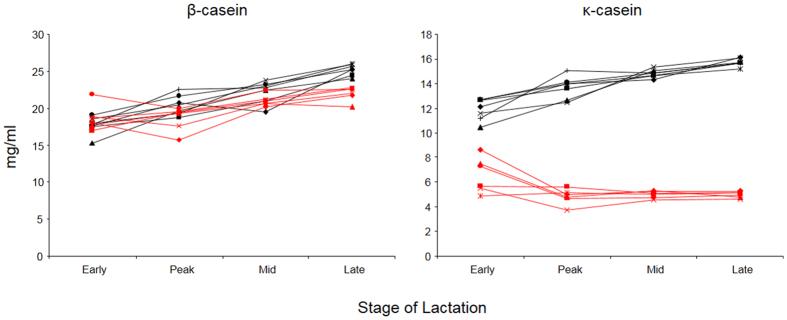
Concentration of total β-casein and κ-casein in TG3 and control milk. Shown are the β-casein and κ-casein levels found in a 24 h milk sample of seven transgenic TG3 (black) and six wild type control (red) cows at early, mid, peak and late lactation.

Natural lactation milk samples from seven TG3 and six control cows were analyzed by high-performance liquid chromatography (HPLC) to quantify the levels of β-casein and κ-casein produced in the milk of these animals. In the TG3 line the normal gene dosage of two endogenous copies for β-casein has been increased by approximately five extra transgene copies^16^. Despite this, we detected only a small increase in total β*-*casein in the TG3-derived milk samples compared with control samples (Fig. 2). In early lactation samples, there was no apparent difference in the β–casein levels. A slight difference became noticeable in milk collected at peak and mid lactation, and was most prominent in late lactation samples with an approximate 15% (P < 0.001) increase in β-casein in the TG3-derived milk compared with control milk.

In addition to the increased gene dosage for β-casein, theTG3 line contains four additional κ-casein encoding transgenes controlled by the regulatory sequences of the more highly expressed β-casein gene to drive the expression of this casein^16^. In contrast to the moderate effect of the increased gene dosage for β-casein, we detected a striking difference in total κ-casein levels between TG3 and control samples. At early lactation, we determined a close to twofold (180%) increase in κ-casein for the TG3 milk samples which further increased to threefold in milk collected at peak (291%), mid (297%) and late lactation (315%, Fig. 2).

### Effect of the high κ-casein content on the size of the casein micelles

**Figure 3:**
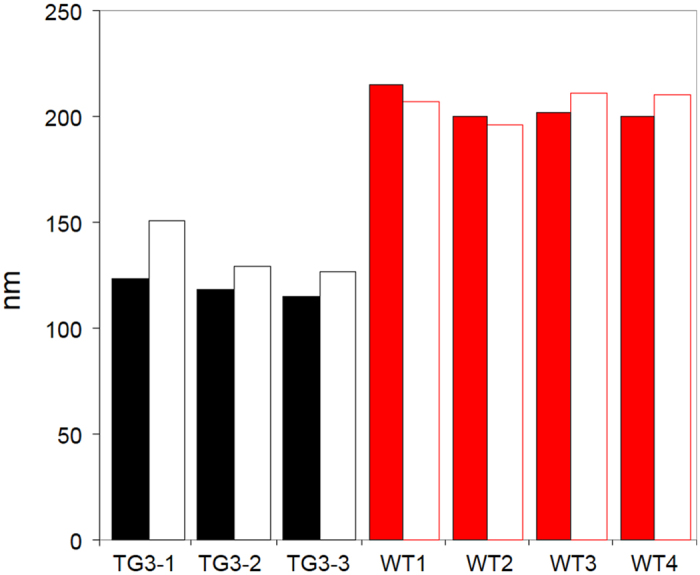
Casein micelle diameter in milk from transgenic and control cows. The size of the casein micelles was measured by photon correlation spectroscopy in whole milk (solid bars) and skim milk (open bars) produced in a natural lactation by three transgenic (TG3-1, -2, -3; black) and four wild type control (WT-1, -2, -3, -4; red) cows.

According to models of casein micelle structure^18^ and transgenic mouse models overexpressing κ-casein^19^,^20^, the concentration of κ-casein is inversely correlated with the size of the micelle. To assess whether this effect is also the case in cattle, we determined the size of the casein micelles for whole milk and skim milk samples derived from three TG3 cows and four control cows using photon correlation spectroscopy. As predicted, the higher κ-casein concentration in the TG3 milk resulted in smaller casein micelles in both, whole milk (P < 0.001) and skim milk (P < 0.005) (Fig. 3). The mean size of the protein particles in the TG3 milk samples were 119 ± 2.3 nm (mean ± standard error of the mean) in whole milk and 136 nm ± 7.7 in skim milk. The micelles present in TG3 milk were on average 85 nm (whole milk) and 70 nm (skim milk) smaller than that from wild type controls (204 ± 3.6 nm and 206 ± 3.4 nm, respectively), representing a size reduction to approximately 58% (whole milk) and 66% of the mean casein micelle size found in wild type milk.

### Effect of the increased gene dosage for β- and κ- casein on the major milk components

**Figure 4:**
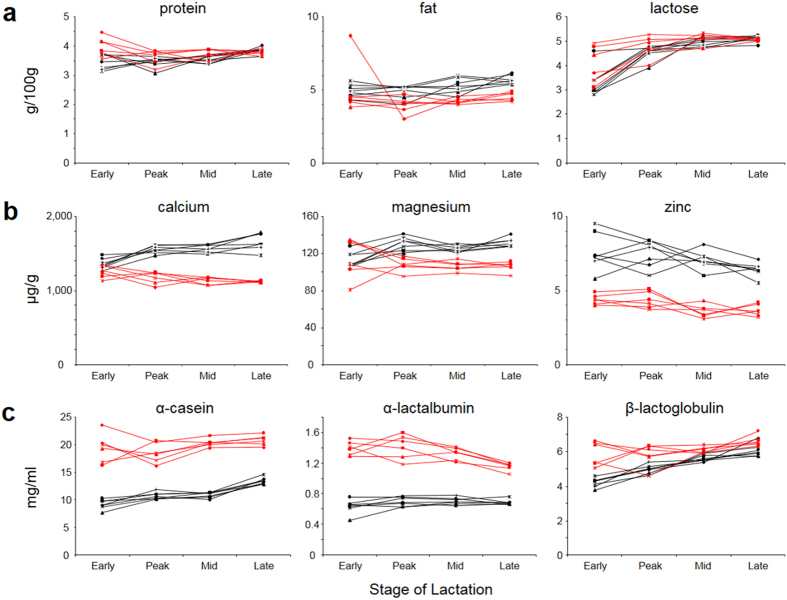
Concentration of major milk components in milk from transgenic and control cows. Shown are the results for the milk components (**a**) protein, fat and lactose; (**b**) the minerals calcium, magnesium and zinc and (**c**) the endogenous milk proteins α-casein, α-lactalbumin and β-lactoglobulin in milk samples from seven transgenic TG3 (black) and six wild type control (red) cows at early, mid, peak and late lactation.

Because the regulation of individual milk components can be interrelated we next wanted to analyze if the activity of the casein transgenes has any overall effect on the major milk components; protein, fat lactose and minerals. Despite the differences in individual proteins described above, total protein levels were the same in TG3 and control milk (Fig. 4a), with the exception of the early milk samples where the total protein content of the milk from TG3 cows (n = 7) was slightly lower than it was in the control milk (n = 6; on average 87%, P = 0.007). On the other hand, addition of extra genes for the two milk proteins β- and κ-casein resulted in a significant increase in total milk fat. Elevated fat levels in the TG3 milk were detected throughout lactation and were approximately 29% (peak, P = 0.004), 25% (mid, P = 0.0008) and 25% (late, P < 0.001) above the values detected in the corresponding control samples (Fig. 4a). Due to an unusually high reading from one of the early lactation control samples there was no significant difference at that stage of lactation. An analysis of 21 minerals present in milk from the transgenic and control animals showed significant increases for some of the most beneficial cations for human nutrition (Fig. 4b). The calcium content of the TG3 milk was increased by up to 49% (late lactation) compared to the controls. The less abundant divalent cations magnesium (Fig. 4b) and strontium (not shown) were found at elevated levels of up to 196% and up to 150% of the control values, respectively. Levels of the minor element zinc were also elevated in TG3 milk (up to 125% of the control values) (Fig. 4b). For two minerals the levels were slightly decreased at mid and late lactation. Potassium in TG3 milk was reduced to 79% (P < 0.001, mid lactation) and 82% (P < 0.001, late lactation) and inorganic phosphate to 86% (P < 0.001, mid lactation) and 93% (P = 0.0045, late lactation) in comparison to the control milk. Other minerals analyzed were unchanged between the samples. The full data set of the standard panel of elements has been included as supplementary information (Table S1). Finally, in contrast to the effect on milk fat and minerals, the level of the major carbohydrate component in milk, lactose, was similar in TG3 and control milk samples (Fig. 4a).

### Effect of the increased gene dosage for β- and κ- casein on the expression levels of other milk proteins

The lack of any effect of transgene expression on the total protein concentration suggests that the expression of the transgenes might occur at the expense of reduced expression levels for endogenous milk proteins. To investigate any such compensatory changes we determined the expression levels of all the major milk proteins in milk from the TG3 and control animals. This revealed marked changes in the expression levels of at least two of the endogenous milk proteins while others were only slightly affected (Fig. 4c). In TG3-derived milk samples the α-casein content was reduced to approximately half the levels found in control milk in early, peak and mid lactation. This difference in the α-casein content was smallest in late lactation samples due to a slight increase of α-casein in late lactation TG3 milk, with TG3 samples containing about 65% of the α-casein found in control milk. Similarly, we also detected a reduction for the whey protein α-lactalbumin in TG3 milk to half the levels found in control milk in early, peak and mid lactation samples. As with α-casein, the difference narrowed, in this case due to a decrease in α-lactalbumin levels in the wild type milk towards late lactation. Late lactation TG3 samples contained 60% of the average α-lactalbumin present in control samples. The whey protein β-lactoglobulin was the least affected milk protein. At early lactation, average β-lactoglobulin levels in TG3 samples were reduced to 70% (P < 0.001) of the control values. With progression of lactation, β-lactoglobulin levels in TG3 samples increased relative to controls (87%, peak lactation; 92%, mid and late lactation). However, only the difference between mid-lactation TG3 and control samples remained significant (P < 0.001).

### Effect of the increased gene dosage for β- and κ- casein on the fatty acid composition of milk fat

The observation that the overexpression of casein genes impact on the fat content of milk prompted us to look in greater detail at the lipid component of the milk, specifically the fatty acid composition. (Fig. S1). Compared to the milk of non-transgenic control cows (n = 7), the milk from transgenic cows (n = 7) had lower proportions of the short (C_6:0_, minus 20%) and medium chain fatty acids (C_8:0,_ minus 38%; C_10:0,_ minus 50%; C_12:0,_ minus 50%; C_14:0,_ minus 30%), and higher proportions of palmitic acid (C_16:0_, plus 25%). In contrast, to the *de novo* synthesized short and medium chain fatty acids by the mammary gland, the longer chain fatty acids (C18 to C22), which are exclusively derived from blood plasma fatty acids of dietary origin or from mobilization of body fat stores, were not affected (Fig. S1, Table S2).

### Effect of the changed composition on milk yield

The volume of milk yield obtained daily or across a full lactation is an important economic parameter which may be affected by the introduction of the additional casein genes and resulting changes to the milk composition. Comparison of the overall milk yields from the seven TG3 and six control cows revealed no significant differences between the transgenic and control animals (Table S3). The total milk volumes produced during the average lactation period of 275 days for the TG3 cows was 3286 L. Because two of the wild type control cows were late calvers, the average lactation period was slightly shorter (226 days) compared to the transgenic cows and resulted in a total production of 3050 L milk. Although the average daily milk yield for the control cows (13 L) was slightly greater than the average milk yield for the TG3 cows (12 L), this difference was not significant. Thus, the transgene-induced production of milk proteins and changes of the milk composition does not affect the milk volume produced by the mammary gland.

### Effects on different variants and isoforms of milk proteins

**Figure 5:**
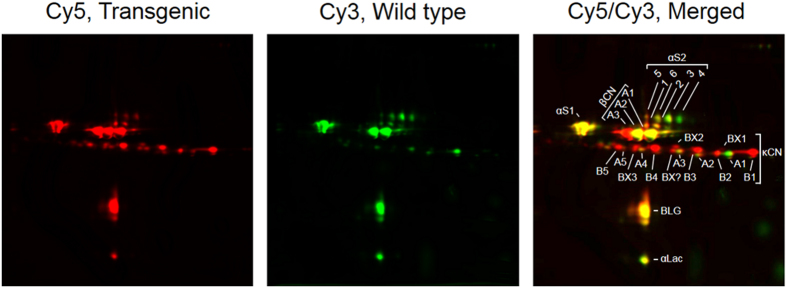
DIGE analysis of milk proteins from transgenic and control cows. Milk from transgenic as well as wild type cows was collected at peak of a natural lactation and the proteins from each cow were individually labelled with Cy5 and Cy3, respectively. Shown is the Cy5 (TG3, red), Cy3 (wild type, green), and a merged color-coded C5/Cy3 signal obtained from a section of a representative gel loaded with WT and TG3 milk. The different isoforms of the milk proteins that were quantified have been labelled in the merged panel. αS1: αS_1_-casein; αS2: αS_2_-casein, isoforms 1–6; βCN: β-casein, isoforms A1-A3; κCN: κ-casein, isoforms A1-A5, B1-B5, BX1-BX3, BX?; αLac: α-lactalbumin; BLG: β-lactoglobulin. The gels are cropped for improved clarity and the full size gels are presented in Supplementary Fig. S3.

To analyze the effect of the β- and κ-casein transgenes on the milk proteins in more detail, including differences of the variant forms and post-translationally modified isoforms of individual milk proteins, we performed a quantitative analysis of peak lactation milk samples using 2D difference in gel electrophoresis (DIGE; Fig. 5). The identity of the separated protein spots for the different milk proteins were confirmed by MS analysis (Supplementary Table S4) and a summary of the DIGE quantification results is documented in Supplementary Table S5. Three distinct variants of β-casein were resolved, corresponding to the A1, A2 and A3 phosphorylated isoforms of the protein. The phosphorylation status of these proteins is not variable, generating only one isoform for each of the β-casein variants. This revealed that in TG3 milk, the endogenous β-casein variant A1 was expressed at only 68% (P < 0.006) of the levels found in control milk whereas the level of the second endogenous β-casein variant A2 was unchanged compared with the wild type control. In contrast to β-casein, κ-casein presented a much more complex pattern with more than ten differently modified isoforms. We concentrated on five previously characterised isoforms^21^ of the endogenous κ-casein A variant which were clearly separated and readily identifiable, comprising the main, most basic isoform modified by one phosphate group, and four glycosylated forms that were further modified by covalent attachment of increasing numbers of a specific tetrasaccharide (Fig. 5, κ-A1-A5). The κ-casein A protein variant whose abundance was most affected by expression of the transgenes was the main, non-glycosylated isoform (κ-A1) which was present at only 42% (P < 0.001, merged signal predominantly green) of the level determined for the control milk. In addition, one of the glycosylated isoforms (κ-A3) was reduced by 29% (P < 0.007) while the isoform κ-A4 showed an increase to 177% (P < 0.049). The other two glycosylated isoforms, κ-A2 and κ-A5, remained unchanged. For αS2-casein, we identified and quantified six isoforms (αS2 1–6), which most likely differ in their extent of phosphorylation and glycosylation. The main difference we observed between the TG3 and control samples was a significant shift in the relative abundance of the six isoforms (Fig. 5). The two least modified, more basic isoforms αS2 4 and αS2 3 were reduced to only 6% (P < 0.001) and 20% (P < 0.001) of the wild type (WT) levels), respectively. A strong reduction (to 34% of WT levels, P < 0.001) was also observed for isoform αS2 6, resulting in green signals in the merged 2D map for these isoforms. In contrast, the most acid, highly phosphorylated and glycosylated isoform αS2 1 in TG3 milk was increased (166%, P < 0.001) in comparison with control milk samples, generating a red signal in the merged 2D map.

Analysis of quantitative differences of the total amounts of the milk proteins was consistent between the DIGE (Table S5) and HPLC (Figs 2 and 4c) analyses. Total β-casein, comprising the endogenous variants A1 and A2 and the transgene derived variant A3 appeared to be slightly increased (24%, P = 0.045) compared with the wild type controls. The high expression levels of the transgene derived κ-casein B variant resulted in a more than fourfold (P < 0.001) increase in total κ-casein, despite the reduced levels in κ-casein A produced by the two endogenous gene copies. The level of αS1-casein, which is normally the most abundant casein, was decreased to 63% of the levels in non-transgenic milk (P < 0.001) and total αS2-casein and α-lactalbumin levels in the TG3 milk reached only 46% (P < 0.001) and 48% (P < 0.001) of the levels in non-transgenic milk, respectively. Consistent with the HPLC results, the DIGE analysis showed a slight reduction in the whey protein β-lactoglobulin in the TG3 milk (78% of WT, P = 0.008) compared with wild type milk

### Effects on the sialic acid content of milk

**Figure 6:**
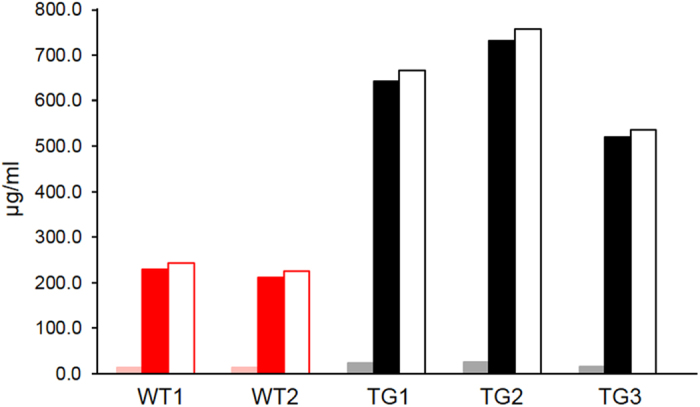
Sialic acid content in milk from transgenic and control cows. Shown is the level of sialic acids associated with milk proteins from two wild type (WT1, WT2) and three transgenic cows (TG3 1–3). Neu5Gc is represented as solid bars in light red and grey, Neu5Ac as red and black solid bars and total sialic acid as red and black open bars for wild type and transgenic samples, respectively.

Milk contains high levels of sialic acid, a significant proportion of which is conjugated to the carbohydrate chains of κ-casein^22^. Moreover, dietary sialic acid has been suggested to be beneficial to brain development^23^. We therefore determined whether the increased abundance of κ-casein also leads to a higher concentration of this potentially health-promoting substance in the transgenic milk. The abundance of the two most common sialic acid groups, N-Glycolyl-D-neuraminic acid (Neu5Gc) and N-Acetyl-D-neuraminic acid (Neu5Ac) as well as total sialic acid were measured in milk proteins from the TG3 and control cows. The results (Fig. 6) showed a 2.9 fold increase (P = 0.01) of Neu5Ac in the milk from the TG cows (632.1 ± 61.8 μg/ml; n = 3) compared to wild type (220.4 ± 8.7 μg/ml; n = 2). Although all Neu5Gc values for the TG samples where above the measured levels for the wild type samples, the average increase of 1.6 fold did not reach a 95% significance level with the limited numbers of samples. The total sialic acid concentration was also increased by 2.8 fold (P = 0.015; 653.4 ± 64.6 μg/ml versus 233.7 ± 9.2 μg/ml). The most heavily glycosylated protein in milk is κ-casein, for which the protein levels are increased several-fold in milk from the TG cows, as described above. The sialic acid content of the milk per mg of κ-casein (46.9 μg/mg for WT, 41.88 μg/mg for TG3) was not significantly altered between the milk from wild type and TG cows (P = 0.37), suggesting that the increase in sialic acid content is consequent upon the high abundance of κ-casein.

## Discussion

The detailed analysis of milk from cows expressing additional copies of β- and κ-casein has shown that the expression of these transgenes resulted in changes in individual milk proteins and micelle size but did not affect the total protein concentration. This is consistent with our previously reported analysis of mammary secretion in pre-pubescent cows after hormonal induction^16^. Competition for expression between exogenous and endogenous milk proteins had also been observed in mice^24^. Production of the ruminant-specific whey protein, β-lactoglobulin in transgenic mice was produced at the expense of endogenous milk proteins. In β-casein knockout mice, a similar compensatory effect had been reported where the absence of β-casein was partly compensated for by an increase of other endogenous milk proteins^25^.

The lack of an effect on total milk protein concentration contrasts with a number of earlier studies altering milk composition by transgenic means. High level expression of human α_1_-antitrypsin in the mammary glands of sheep produced no change in expression of the endogenous milk proteins^26^. In another study, expression of human lactoferrin and lysozyme did not alter the abundance of endogenous milk proteins^27^. On the other hand, expression of human butyryl-cholinesterase under the control of the goat β-casein promoter lowered casein levels without altering total protein concentration^28^.

Our results indicate that, at least for bovine, there might be a biological ceiling for the protein output of the mammary gland. Whether this is a consequence of strict regulatory mechanisms or could be overcome by an increased feed intake needs further investigation.

The differences we observed in the abundance of individual isoforms of the endogenously expressed casein genes, as well as differences in their post-translational modifications suggest that in the cow at least, there appears to be a mechanism that controls the balance of some of the casein isoforms but not others, and that perturbation of this results in a cascade of compensatory changes. A possible purpose for the existence of such a mechanism is to maximise incorporation of the caseins into the micellular structure, since these proteins have limited solubility in free solution. Further investigation is required to determine to what extent these effects are specific to the promoter system to drive expression or are generic responses to perturbation.

The marked difference in the overall abundance of κ-casein, as well as its state of post-translational modification, are likely to be the cause of the decreased size of the micelles in the milk from the transgenic cows, since κ-casein plays a key role in maintaining the micelle structure^29^. The smaller micelle size in turn is likely to be responsible for the reduced opacity of the transgenic milk, as well as the altered amino acid composition of cheese produced from the transgenic milk, which we reported previously^17^. The increased levels of sialic acid that we observed in the transgenic milk compared with controls is likely to be a secondary effect consequent on the increased abundance of κ-casein, to which the majority of the sialic acid in milk is bound. This observation shows that the bovine mammary gland is able to produce a higher than normal amount of sialic acid when presented with an increased amount of substrate, apparently without exceeding its capacity to synthesise this sugar group. The reason for the increased level of calcium, magnesium and strontium in the transgenic milk is not certain, but could possibly also be the consequence of an altered micelle structure, since at least some of these elements are known to be associated with the micelle^30^.

Although the level of milk fat present in milk varies widely among species, it is relatively constant within a given species. About half of the fatty acids, restricted to the long chain fatty acids, are derived from blood plasma lipids of dietary origin and to a lesser extent from mobilisation of body lipid stores^31^,^32^. This explains why milk fat composition can be highly variable depending on the diet and the nutritional status^33^,^34^. Because all cows in this study shared the same genetic background and were farmed together, the observed differences in the fatty acid composition must have been generated by *de novo* fat synthesis in the mammary gland. This process is based on a stepwise chain elongation reaction that incorporates two-carbon building blocks to synthesise the end product palmitic acid (C16:0). The short to medium fatty acids (C4:0-C14:0) are almost exclusively *de novo* synthesized in the mammary gland while only about half of the C16 fatty acids are generated *de novo*^35^. The remainder of C16 plus all long chain fatty acids are derived from the diet or body fat mobilization. We observed a decrease in the short to medium fatty acids and an increase of C16:0 in the TG3 milk, which is consistent with a higher degree of completion of *de novo* fat synthesis in the mammary gland. This enhanced *de novo* milk fat synthesis as a result of overexpressing casein genes highlights the complex molecular mechanisms controlling milk composition. It is likely that the casein overexpression puts the secretory machinery of the mammary gland under additional pressure. Conceivably, this may slow the secretion process and thereby increase the opportunity for the involved enzymes to extend the fatty acid chain, boosting *de novo* milk fat synthesis. This could result in an altered balance of transcription factor-promoter interactions among the milk protein and lipid synthesis genes, which in turn could lead to alterations in the relative rates of transcription of these genes. However, these ideas are mere speculation at this time, and further studies will be required to confirm the underlying mechanisms driving the altered milkfat production. Whether the changes in total fat and the levels of different monounsaturated fatty acids balance out or may provide an overall health benefit needs further investigation.

The findings presented here have relevance to the future uses of transgenically altered milk. Production of protein-based pharmaceuticals through their secretion into milk is one such possibility. Our study has shown that the use of a relatively strong mammary-specific promoter based on a major milk protein can be used to boost expression levels well beyond that of the less abundant milk proteins such as lactoferrin or lysozyme. A better understanding of the mechanisms governing the balance of the individual proteins in milk could well lead to even more efficient production of transgenes and their extraction from milk, if expression of non-essential endogenous milk proteins can be minimised. Commercial processing of transgenic milk for dairy production is another area of relevance. For this application, the study has made a number of relevant observations. Casein is of high commercial value because it makes up about 80% of all milk protein and is the protein fraction that is captured in cheese. A smaller micelle size such as was observed here, is associated with increased thermal stability during processing and trapping of more milk solids in cheese manufacture, further increasing cheese yield. Although the possible savings in processing due to improved thermostability and gains in casein and cheese yield as described here may appear small, the scale of international dairy industry operations amplifies such changes into enormous additional values^16^,^36^. Also, some of the secondary alterations, for example increased essential amino acids, micronutrients and sialic acid, could provide a health benefit and additional value to dairy products. The study underscores the need to better understand how promoter usage effects milk characteristics as a whole.

The findings in this study have demonstrated the feasibility of altering milk characteristics through a transgenic approach, and also revealed some of the secondary alterations that can occur and that should be considered in undertaking such an approach. The high casein milk produced by the TG3 transgenic cows has distinctly altered characteristics that could provide improved dairy processing characteristics and additional nutritional health benefits. Thus, this novel milk provides a valuable model to investigate the impact of milk composition and identify new opportunities on how to optimize cows’ milk for human consumption.

## Materials and Methods

All experiments involving animals were done in compliance with NZ laws and were approved by the NZ Environmental Protection Agency and predecessor organisation and the Ruakura Animal Ethics Committee.

The study involved seven transgenic founder cows from theTG3 line, and six age matched control cows that were produced in parallel from the unmodified parental cell line and thus were genetically identical to TG3 cows except for the transgene insertions. Detailed procedures for their production are described elsewhere^16^. All cows were mated by artificial insemination with the same bull to induce them into a natural lactation following calving.

### Milk collection

Cows were machine milked twice daily and milk was sampled for subsequent analyses at four different stages during the lactation: early lactation (between day 17 and 25 of lactation), peak lactation (between day 50 and 72 of lactation), mid lactation (between day 120 and 142 of lactation) and late lactation (between day 200 and 250 of lactation). Milk samples taken from the afternoon milking and the morning milking of the following day were combined to generate a representative milk sample of a 24 h period. Milk yield was determined from milk volume data automatically collected (DeLaval, Sweden) for each cow at each milking throughout the course of the lactation.

### Quantification of major milk components and individual milk proteins

Total protein, lipids and lactose of milk samples were determined according to standard analytical methods^37^. Briefly, total protein was deduced from total nitrogen following flash combustion using a Carlo Erba NA 1500 Analyser (CE Instruments, Milan, Italy), total lipids were measured by diethyl ether extraction using a Soxhlet apparatus and lactose content in milk was quantified by HPLC following the extraction with ethanol. For mineral analysis, milk samples were acid digested and evaluated with an inductively coupled plasma atomic emission spectrometer (Applied Research Laboratories, Dearborn, MI; simultaneous emission spectrometer 34000) as described previously^17^. The concentration of the major milk proteins in skimmed milk was determined by reversed-phase HPLC against commercial milk protein standards^38^.

### Micelle size determination

Whole milk and skim milk samples were incubated for 10 minutes at 35 °C, inverted to ensure a homogenous solution and diluted to a concentration of 0.02 mL/mL in 5 KDa tangential flow ultrafiltration permeates prepared from WT and TG3 skim milk, respectively. The mean size of casein micelles was determined as the average of three measurements by photon correlation spectroscopy using a Zetasizer 4 (Malvern Instruments, UK).

### Fatty acid analysis of milk fat

Milk fat was extracted with a mixture of hexan and isopropanol^39^. After evaporating the solvent, the milk fat extracts were resuspended in hexan and derivatized by transmethylation into methyl esters for quantification and identification by gas chromatography with flame ionization detection against known standards^40^.

### Analysis of milk proteins by 2D electrophoresis and DIGE

2D gel electrophoresis of milk proteins and DIGE analysis was performed on skimmed milk as previously described^41^,^42^,^43^. Milk samples were collected during a period of peak natural lactation from seven transgenic cows, three ‘isogenic’ control cows (wild type) and a wild type control cow with a κ-casein A/B genotype. Each transgenic milk sample was minimally labelled with Cy5 in separate reactions, while each wild type milk was minimally labelled with Cy3. A pooled internal standard, comprising equal aliquots from each of the eleven WT and transgenic milk samples, was minimally labelled with Cy2. The dyes were switched between the transgenic and wild type milk groups to facilitate reverse-labelling of the samples in order to eliminate bias. A total of eight 2D gels were each loaded with 30 μg of a mixture comprising an individual Cy5-labelled transgenic milk, an individual Cy3-labelled WT milk and an aliquot of the Cy2-labelled pool as internal control. A further eight 2D gels were each loaded with the reverse labelled transgenic and WT milk samples. The pairwise comparisons within each of the eight gels were WT1 with TG1–3, WT2 with TG4–6 and WT3 with TG7 and a natural κ-casein A/B control. Each mixture was subjected to 2D electrophoresis using pH 4–7 Immobiline™ DryStrip gels (18 cm; GE Healthcare, USA) as the first dimension, and 12.5% SDS polyacrylamide gels as the second dimension. Following SDS-PAGE, the gels were scanned immediately at 100 μm resolution using a Molecular Imager^®^ FX™ (Bio-Rad, CA, USA) in multiple channel mode. Image analysis was performed using PDQuest v7.3 (Bio-Rad). Spot patterns were matched and data were normalized using the ‘in-buit’ local regression model (LOESS). Spot volumes were log transformed and quantified relative to the internal standard Cy2 signal, which served as a normalisation control for unequal loading as well as variations in sensitivity of detection between gels. A mean ratio of TG/WT was produced for each spot and the paired Students t-test was performed at the 95% significance level to determine which proteins were differentially expressed between wild type and transgenic milks. Individual protein spots were excised and subjected to tandem LC MS to confirm their identity (see Supplementary Materials for further details). The identity of each spot was also cross-checked against the individual casein isoforms identified previously by the 2D analysis of Holland and co-workers^21^.

### Quantification of the sialic acid content in milk

The quantification of the sialic acid content in milk was determined following the method as described^44^. Briefly, sialic acids were hydrolyzed from glycoconjugates, derivatized using 1,2-diamino-4,5-methylenedioxybenzene dihydrochloride, and separated using reversed-phase HPLC^44^. Known standards of Neu5Ac and Neu5GC were used to quantify the sialic acid abundance in milk samples.

### Statistical analyses

Statistical significance levels presented as P values were determined by two-tailed T-tests. The average of experimental data were expressed as mean plus and minus standard error of the mean.

## Additional Information

**How to cite this article**: Laible, G. *et al*. Increased gene dosage for β- and κ-casein in transgenic cattle improves milk composition through complex effects. *Sci. Rep.* **6**, 37607; doi: 10.1038/srep37607 (2016).

**Publisher’s note:** Springer Nature remains neutral with regard to jurisdictional claims in published maps and institutional affiliations.

## Supplementary Material

Supplementary Information

Supplementary Table S1

Supplementary Table S1
