# Supplementary material for: Increased gene dosage for β- and κ-casein in transgenic cattle improves milk composition through complex effects
Source: Sci Rep. 2016 Nov 23;6:37607. doi: 10.1038/srep37607 (PMC5120311; doi:10.1038/srep37607)
Supplement: Supplementary Information [file srep37607-s1.pdf]

Increased gene dosage for β- and κ-casein in transgenic cattle improves milk composition through complex effects

Götz Laible, Grant Smolenski, Thomas Wheeler, Brigid Brophy

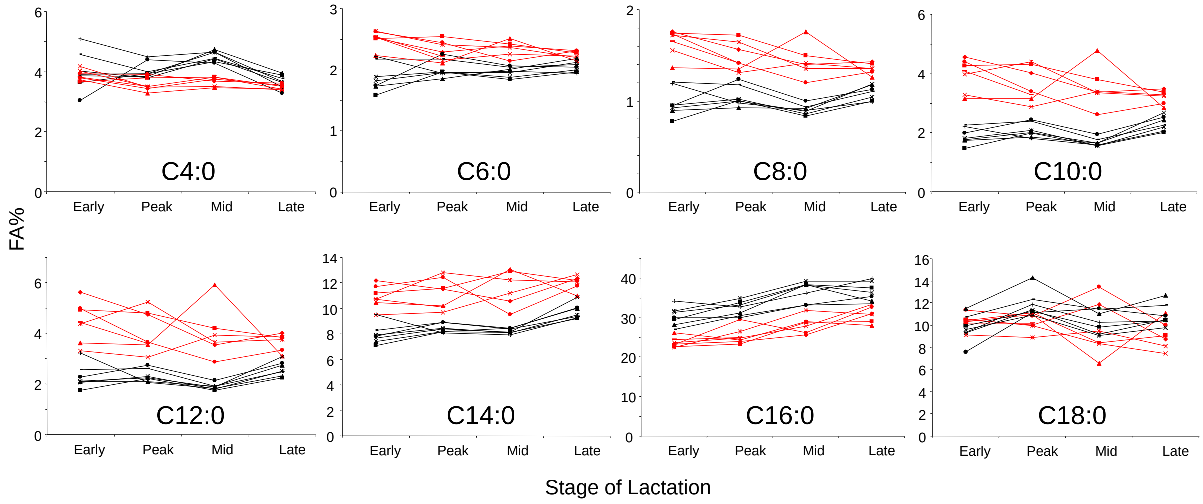

Figure S1: Fatty acid composition in milk fat from transgenic and control cows.

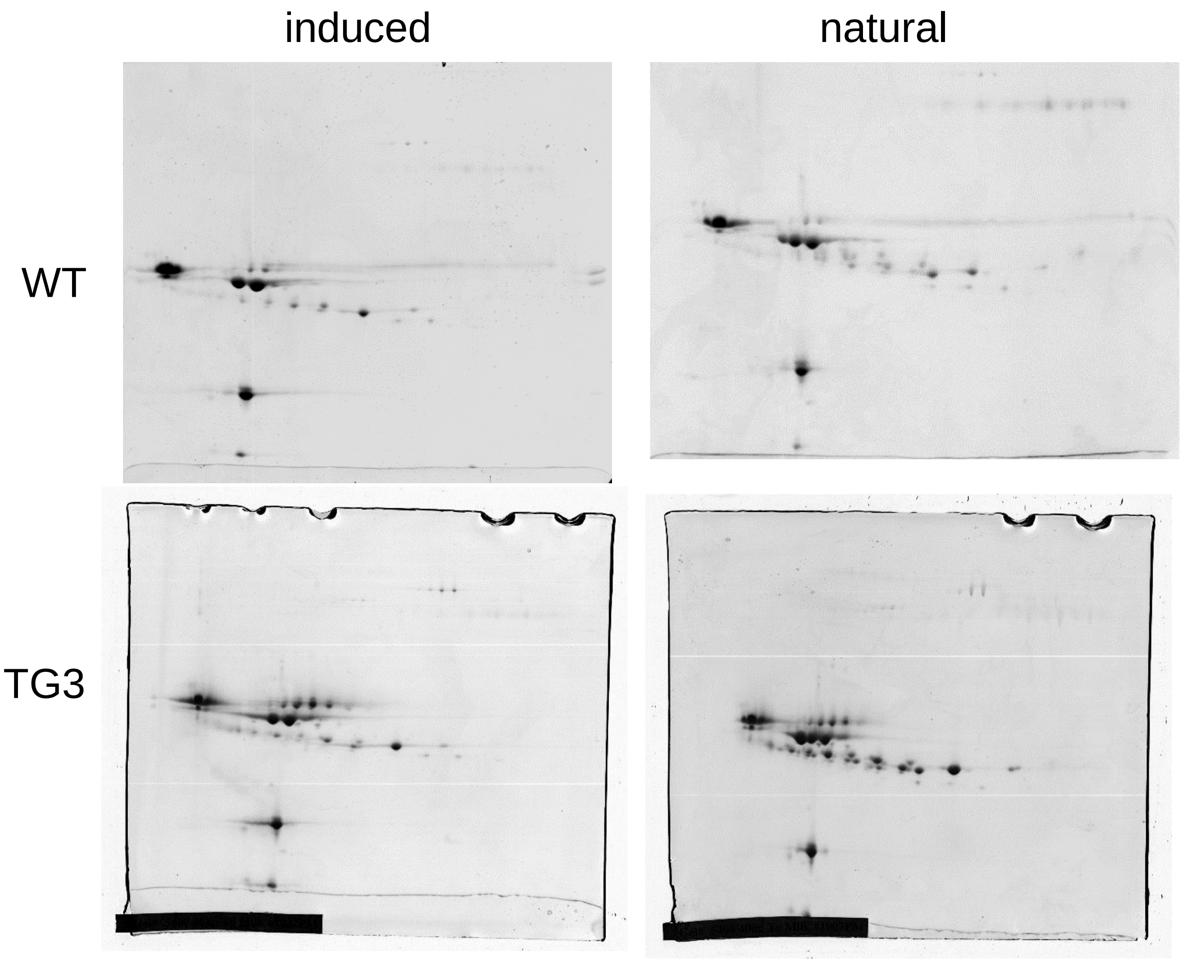

Figure S2: Full size gels used for the two-dimensional milk protein analysis documented in Fig. 1.

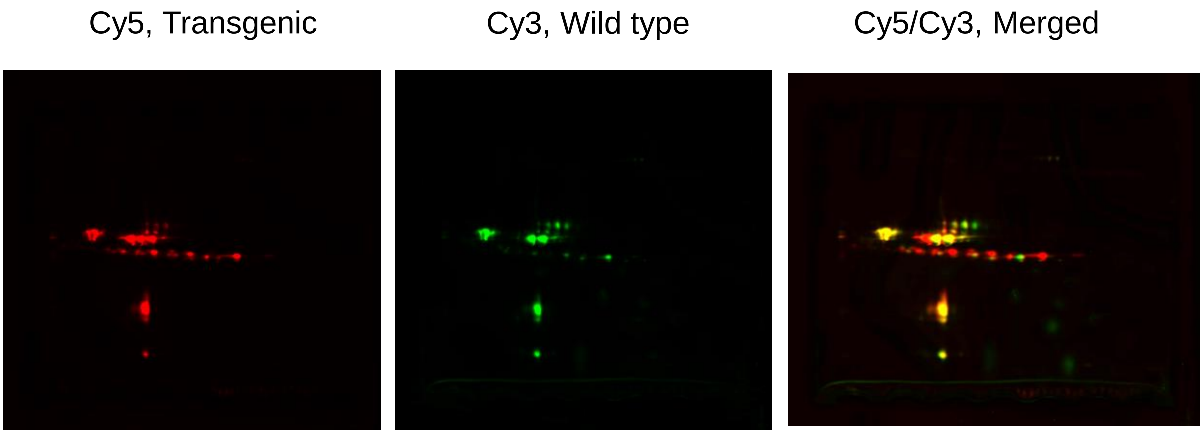

Figure S3: Full size gels used for the DIGE analysis of milk proteins shown in Fig. 5.

Table S3: First lactation milk yields of transgenic and wild type cows.

Cow	Sum of Milk, am	Sum of Milk, pm	Sum of Milk Total	Days in milk	Avg. daily milk
TG1	2383	1216	3967.85	276	14.4
TG2	1960	970	3234	276	11.7
TG3	1799	958	3043.45	280	10.9
TG4	2257	1131	3729	281	13.3
TG5	1288	730	2217.05	278	8.0
TG6	2201	1152	3719.9	260	14.3
TG7	1846	954	3093.45	275	11.2
WT1	1741	958	2957.85	256	11.6
WT2	2223	1219	3833	273	14.0
WT3	862	567	1530.5	171	9.0
WT4	1617	958	2714.4	160	17.0
WT5	2577	1396	4378.1	277	15.8
WT6	1663	918	2886.64	219	13.2
TG, avg.	1962	1016	3286	275	12.0
WT, avg.	1504	861	2577	226	13.0
P value, (TG vs. WT)	0.51	0.92	0.60	0.03	0.33

Table S4: Summary of MS identification of individual protein spots generated by 2D separation of milk proteins.

DIGE code	Protein identification	Avg. mass (Da)	# peptides	# unique peptides	Coverage	Phosphorylation
αS2-1	α-S2-casein	26019	6	6	29	Ser^{46}, Ser^{158}
αS2-2	α-S2-casein	26019	11	11	48	Ser^{46}, Ser^{144}, Thr^{155}, Ser^{158}
αS2-3	α-S2-casein	26019	14	14	50	Ser^{46}, Ser^{144}, Thr^{155}, Ser^{158}
αS2-4	α-S2-casein	26019	11	11	38	Ser^{158}
αS2-5	α-S2-casein	26019	14	14	48	Ser^{46}, Ser^{158}
αS2-6	α-S2-casein	26019	13	13	45	Ser^{46}, Ser^{158}
βCN-A1	β-casein (variant A1)	25076	10	4	67	Ser^{50}
βCN-A2	β-casein (variant A2)	25107	10	4	62	Ser^{50}
	β-casein (variant A3)	25107	10	4	62	Ser^{50}
βCN-A3	β-casein (variant A3)	25098	10	3	40	Ser^{50}
	β-casein (variant A2)	25107	10	3	40	Ser^{50}
βLG	β-lactoglobulin	19883	20	20	68	
αLac	α -lactalbumin	16247	3	3	23	
κCN-A1	κ-casein (variant A)	21269	5	1	66	Thr^{138}
κCN-A3	κ-casein (variant A)	21269	16	1	66	Thr^{138}
κCN-A4	κ-casein (variant A)	21269	11	0	45	
	κ-casein (variant B)	21237	11	0	45	
κCN-B1	κ-casein (variant B)	21237	15	0	51	
	κ-casein (variant A)	21269	15	0	51	
κCN-B2	κ-casein (variant B)	21237	9	0	35	
	κ-casein (variant A)	21269	9	0	35	
κCN-B3	κ-casein (variant B)	21237	19	0	54	
	κ-casein (variant A)	21269	19	0	54	
κCN-B4	κ-casein (variant B)	21237	26	0	53	
	κ-casein (variant A)	21269	26	0	53	
κCN-B5	κ-casein (variant B)	21237	12	0	47	
	κ-casein (variant A)	21269	12	0	47	

DIGE code	Protein identification	Avg. mass (Da)	# peptides	# unique peptides	Coverage (%)	Phosphorylation site
κCN-BX1	κ-casein (variant B)	21237	2	0	15	
	κ-casein (variant A)	21269	2	0	15	
κCN-BX2	κ-casein (variant B)	21237	11	0	41	
	κ-casein (variant A)	21269	11	0	41	
κCN-BX3	κ-casein (variant B)	21237	6	0	35	
	κ-casein (variant A)	21269	6	0	35	
κCN-BX?	κ-casein (variant B)	21237	2	0	11	
	κ-casein (variant A)	21269	2	0	11	

Table S5: Quantification of selected DIGE spots for separated isoforms of the main milk proteins and their variants in TG3 and WT milk.

Protein<br>Spot	Cy3 (WT)<br>Abund.	SE	Cy5 (Tg)<br>Abund.	SE	Fold change<br>Cy5/Cy3	Significance<br>T-test*
β-casein A1	92	8.9	55	4.7	0.68	***0.006***
β-casein A2	77	9.7	82	3.1	1.06	0.506
β-casein A3	0	0	73	20	*de novo*	
β-casein total	170	7.9	208	20.3	1.24	***0.045***
κ-casein A1	26	5.6	11	2.4	0.42	***<0.001***
κ-casein A2	8.3	0.7	8.4	1.2	1.02	0.987
κ-casein A3	16	1.5	12	1.2	0.71	***0.007***
κ-casein A4	11	1.6	20	5.4	1.77	***0.049***
κ-casein A5	8.5	2.0	7.6	1.2	0.90	0.860
κ-casein B1	0	0	51	9.7	*de novo*	
κ-casein B2	0	0	28	2.9	*de novo*	
κ-casein B3	0	0	41	3.2	*de novo*	
κ-casein B4	0	0	50	4.6	*de novo*	
κ-casein B5	0	0	25	2.3	*de novo*	
κ-casein BX?	0	0	12	3.0	*de novo*	
κ-casein BX1	0	0	11	1.9	*de novo*	
κ-casein BX2	0	0	15	3.0	*de novo*	
κ-casein BX3	0	0	18	4.2	*de novo*	
κ-casein, total	70	6.1	311	19	4.42	***<0.001***
α-S_1 casein	302	15	190	11	0.63	***<0.001***
α-S_2 casein 1	12	1.9	20	1.9	1.66	***<0.001***
α-S_2 casein 2	13	1.4	10	0.55	0.79	0.103
α-S_2 casein 3	45	5.7	8.9	1.5	0.20	***<0.001***
α-S_2 casein 4	8.7	3.3	0.52	0.12	0.06	***<0.001***
α-S_2 casein 5	11	3.3	6.4	0.89	0.57	0.065
α-S_2 casein 6	32	4.8	11	1.3	0.34	***<0.001***
α-S_2 casein, total	122	14	56.8	4.0	0.46	***<0.001***
α-lactalbumin	55	5.8	27	2.5	0.48	***<0.001***
β-lactoglobulin	272	5.2	213	13	0.78	***0.008***

* P values below 0.05 were considered a significant difference and are shown in bold italics. P-values were determined by paired t-test using log transformed spot abundance.

SUPPLEMENTARY MATERIALS AND METHODS

In-gel tryptic digestion:

Protein spots of interest were excised from the 2-DE gel with a disposable scalpel blade and destained as previously described[1]. Following destaining, in-gel tryptic digestion was performed as described below: The gel pieces were dehydrated by the addition of 50 µL 100% acetonitrile and then dried under vacuum. The gel pieces were rehydrated with 10 µL 0.1 mg/mL modified trypsin (Promega, cat #V5111) in 25 mM ammonium bicarbonate, pH 7.8/5% acetonitrile. After 20 min, a further 30 µL of 25 mM ammonium bicarbonate, pH 7.8/5% acetonitrile was added to cover the gel pieces and incubated at 37 °C for 16 h (overnight) at 37 °C. The mixtures were sonicated for 10 min, after which the supernatant was removed and kept. Peptides were then extracted with two 40 µL washes of extraction buffer (40% acetonitrile/0.2% formic acid) in a sonicating water bath, and then finally with 40 µL 100% acetonitrile. The pooled peptide extracts were lyophilised with a vacuum concentrator (SpeedVac), and resuspended in 50 µL 0.2% formic acid/2% acetonitrile for mass spectrometric analysis. Reduction of disulphide bonds with dithiothreitol and alkylation with iodoacetamide was not required for 2-DE spots, as this process had been performed during IPG-strip equilibration.

UHPLC-separation:

An Ultimate 3000 UHPLC system (Thermo Fisher Scientific, Waltham MA, USA) was used for the separation of the protein digests. Buffer A (0.2% formic acid in water) and buffer B (0.2% formic acid in acetonitrile) were used as mobile phases for gradient separation. For each run, 5 µL of protein digest was automatically loaded onto a Thermo Scientific Hypersil Gold C18 column (100 mm × 2.1 mm, 1.9 µm particle size) at 3% buffer B with a flow rate of 0.6 mL/min. The column temperature was maintained at 55 °C. Bound peptides were eluted by linear gradient separation as follows: 3-45% B from 1.5 to 20 min, 45-90% B from 20 to 20.2 min, 90% B from 20.2 to 21.2 min, 90-3% B from 21.2 to 21.5 min, and 3% B from 21.5 to 25 min.

Mass spectrometry:

Peptides eluted from the column were analysed in data-dependent MS/MS mode on a qExactive OrbiTrap mass spectrometer (Thermo Fisher Scientific, Dan Jose, USA) using a heated electrospray ionization (HESI-II) source for ionization in positive ion mode. The instrument parameters were as follows: the capillary temperature was set to 350 °C, and the electrospray voltage was 4.0 kV. The MS instrument was operated in a top 5 data-dependent acquisition (DDA) mode to automatically switch between full-scan MS and MS/MS acquisition. Fullscan MS spectra (m/z 200–2000) were acquired in the Orbitrap with resolution 70,000 (at m/z 200). Automatic gain control (AGC) target value was 1e6 counts, and the maximum injection time was 100 ms. For tandem MS spectra, the five most abundant precursor ions with charge state ≥2 were fragmented in the HCD collision cell, using an isolation width of 4.0 m/z, a normalized collision energy of 30%, and a mass resolution of 17,500 (at m/z 200). The ion selection threshold was 1e5 counts, and the maximum allowed ion accumulation time was 50 ms. For all analyses, the dynamic exclusion time was set to 15 s.

Database searching:

Raw data files were imported in PEAKS Studio v7.5 (Waterloo, ON, Canada) and searched against an 'in-house' bovine milk protein database that contained separate entries for the three β-casein and two κ-casein isoforms. Database searching parameters included up to two missed cleavages for semi-tryptic digestion, precursor ion mass tolerance 10 ppm, product ion mass tolerance 0.1 Da, cysteine carbamidomethylation as a fixed modification, and oxidised methionine, phosphorylated (STY) and deamidated (NQ) as variable modifications. The peptide false discovery rate was estimated by the decoy fusion method[2] and was set at a maximum of 1%.

REFERENCES:

1 Smolenski G, Haines S, Kwan FY, Bond J, Farr V, Davis SR, Stelwagen K, Wheeler TT. Characterisation of host defence proteins in milk using a proteomic approach. *J. Proteome Res* 6, 207-215, doi:10.1021/pr0603405 (2007).

2 Zhang J, Xin L, Shan B, Chen W, Xie M, Yuen D, Zhang W, Zhang Z, Lajoie GA, Ma B. PEAKS DB: de novo sequencing assisted database search for sensitive and accurate peptide identification. *Mol Cell Proteomics*. 11, M111-010587, doi:10.1074/mcp.M111.010587 (2012).
